# Supplementary material for: End Sequence Analysis Toolkit (ESAT) expands the extractable information from single-cell RNA-seq data
Source: Genome Res. 2016 Oct;26(10):1397–410. doi: 10.1101/gr.207902.116 (PMC5052061; doi:10.1101/gr.207902.116)
Supplement: Supplemental Material [file supp_gr.207902.116_Supp_Materials.tar.gz › ESAT-esat_v0.1/doc/html/classbroad_1_1core_1_1math_1_1_permutation_distribution.html]

ESAT: broad.core.math.PermutationDistribution Class Reference


ESAT


- Main Page
- Related Pages
- Packages
- Classes
- Files

- Class List
- Class Index
- Class Hierarchy
- Class Members

All Classes Namespaces Files Functions Variables Enumerator Pages

- broad
- core
- math
- PermutationDistribution

Public Member Functions |
Static Public Member Functions |
List of all members

broad.core.math.PermutationDistribution Class Reference

|  |  |
| --- | --- |
| Public Member Functions | |
|  | PermutationDistribution (Annotation parentRegion, String bamFile, String bedFile, int numPermutations) throws IOException |
|  | |
|  | PermutationDistribution (Annotation parentRegion, String bamFile, String bedFile, int numPermutations, int maxGenomicSpan) throws IOException |
|  | |
|  | PermutationDistribution (Annotation parentRegion, AlignmentModel alignments, CoordinateSpace coordinateSpace, int numPermutations) |
|  | |
|  | PermutationDistribution (Annotation parentRegion, AlignmentModel alignments, CoordinateSpace coordinateSpace, int numPermutations, int maxGenomicSpan) |
|  | |
| double | getScanPvalue (Annotation window) |
|  | |
| EmpiricalDistribution | getScanDistribution (int windowSize) |
|  | |
| void | writeWigEachPosition (String outWig) throws IOException |
|  | |
| void | writeWindowsWithPvalue (int windowSize, String outBed) throws IOException |
|  | |

|  |  |
| --- | --- |
| Static Public Member Functions | |
| static void | main (String[] args) throws IOException |
|  | |

## Detailed Description

Author
:   prussell

## Constructor & Destructor Documentation

|  |  |  |  |
| --- | --- | --- | --- |
| broad.core.math.PermutationDistribution.PermutationDistribution | ( | Annotation | *parentRegion*, |
|  |  | String | *bamFile*, |
|  |  | String | *bedFile*, |
|  |  | int | *numPermutations* |
|  | ) |  | throws IOException |

Parameters
:   |  |  |
    | --- | --- |
    | parentRegion | Parent region |
    | bamFile | Bam file |
    | bedFile | Bed file for transcriptome space |
    | numPermutations | Number of permutations |

Exceptions
:   |  |  |
    | --- | --- |
    | IOException |  |

Here is the caller graph for this function:

|  |  |  |  |
| --- | --- | --- | --- |
| broad.core.math.PermutationDistribution.PermutationDistribution | ( | Annotation | *parentRegion*, |
|  |  | String | *bamFile*, |
|  |  | String | *bedFile*, |
|  |  | int | *numPermutations*, |
|  |  | int | *maxGenomicSpan* |
|  | ) |  | throws IOException |

Parameters
:   |  |  |
    | --- | --- |
    | parentRegion | Parent region |
    | bamFile | Bam file |
    | bedFile | Bed file for transcriptome space |
    | numPermutations | Number of permutations |
    | maxGenomicSpan | Max genomic span for paired reads |

Exceptions
:   |  |  |
    | --- | --- |
    | IOException |  |

|  |  |  |  |
| --- | --- | --- | --- |
| broad.core.math.PermutationDistribution.PermutationDistribution | ( | Annotation | *parentRegion*, |
|  |  | AlignmentModel | *alignments*, |
|  |  | CoordinateSpace | *coordinateSpace*, |
|  |  | int | *numPermutations* |
|  | ) |  |  |

Parameters
:   |  |  |
    | --- | --- |
    | parentRegion | Parent annotation |
    | alignments | Collection of annotations to count e.g. read mappings |
    | coordinateSpace | Coordinate space |
    | numPermutations | Number of permutations |

|  |  |  |  |
| --- | --- | --- | --- |
| broad.core.math.PermutationDistribution.PermutationDistribution | ( | Annotation | *parentRegion*, |
|  |  | AlignmentModel | *alignments*, |
|  |  | CoordinateSpace | *coordinateSpace*, |
|  |  | int | *numPermutations*, |
|  |  | int | *maxGenomicSpan* |
|  | ) |  |  |

Parameters
:   |  |  |
    | --- | --- |
    | parentRegion | Parent annotation |
    | alignments | Collection of annotations to count e.g. read mappings |
    | coordinateSpace | Coordinate space |
    | numPermutations | Number of permutations |
    | maxGenomicSpan | Max genomic span for paired reads |

## Member Function Documentation

|  |  |  |  |  |  |
| --- | --- | --- | --- | --- | --- |
| EmpiricalDistribution broad.core.math.PermutationDistribution.getScanDistribution | ( | int | *windowSize* | ) |  |

Get scan distribution for the window size

Parameters
:   |  |  |
    | --- | --- |
    | windowSize | Window size |

Returns
:   Scan distribution

Here is the caller graph for this function:

|  |  |  |  |  |  |
| --- | --- | --- | --- | --- | --- |
| double broad.core.math.PermutationDistribution.getScanPvalue | ( | Annotation | *window* | ) |  |

Get scan P value of window

Parameters
:   |  |  |
    | --- | --- |
    | window | The window |

Returns
:   The percentage of permutations with a window of the same size that has more overlapping fragments

Here is the call graph for this function:

Here is the caller graph for this function:

|  |  |  |  |  |  |
| --- | --- | --- | --- | --- | --- |
| static void broad.core.math.PermutationDistribution.main | ( | String[] | *args* | ) | throws IOException |

static

Parameters
:   |  |  |
    | --- | --- |
    | args |  |

Exceptions
:   |  |  |
    | --- | --- |
    | IOException |  |

Here is the call graph for this function:

|  |  |  |  |  |  |
| --- | --- | --- | --- | --- | --- |
| void broad.core.math.PermutationDistribution.writeWigEachPosition | ( | String | *outWig* | ) | throws IOException |

Write a wig file of -log10(Pvalue) at each position of annotation

Parameters
:   |  |  |
    | --- | --- |
    | outWig | Output wig file |

Exceptions
:   |  |  |
    | --- | --- |
    | IOException |  |

Here is the call graph for this function:

|  |  |  |  |
| --- | --- | --- | --- |
| void broad.core.math.PermutationDistribution.writeWindowsWithPvalue | ( | int | *windowSize*, |
|  |  | String | *outBed* |
|  | ) |  | throws IOException |

Write windows to bed file with scan P value in score field

Parameters
:   |  |  |
    | --- | --- |
    | windowSize | Window size |
    | outBed | Output bed file |

Exceptions
:   |  |  |
    | --- | --- |
    | IOException |  |

Here is the call graph for this function:

---

The documentation for this class was generated from the following file:

- java/broad/core/math/PermutationDistribution.java


---

Generated on Fri Aug 15 2014 15:10:53 for ESAT by  

 1.8.7
